# Lung sound amplitude measured by vibration response imaging is influenced by the presence of secretions

**DOI:** 10.1186/cc9593

**Published:** 2011-03-11

**Authors:** S Lev, AS Stern-Cohen, MS Shapiro, JC Cohen, YG Glickman, PS Singer

**Affiliations:** 1 Rabin Medical Center, Beilinson Campus, Petach Tikva, Israel; 2 DeepBreeze Ltd, Or-Akiva, Israel

## Introduction

There is no valid estimation of the presence of airway secretions in mechanically ventilated patients. Secretions may amplify breath sounds by increasing turbulence in the airways or alternatively decrease breath sounds by obstructing air flow. Vibration response imaging (VRI) was recently suggested as a tool to assess secretion removal following physiotherapy [1]. The objective of our analysis was to describe the acoustic effects of secretion removal by measuring the lung sound amplitudes pre and post airway suction in both lungs.

## Methods

Twenty-two recordings pre-suction and 22 recordings post-suction (19 patients) were performed with VRI while the mode of ventilation remained constant. The sound amplitude measurements before and after the suction procedure were compared.

## Results

**Figure 1:**
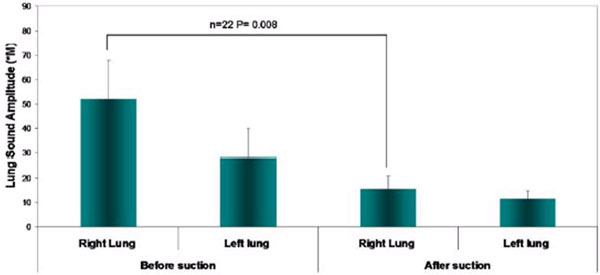
**Lung sound amplitude of secretion removal (mean ± SEM)**.

After suction a decrease in total lung sound amplitude was detected in all of the recordings. The lung sound amplitude of the right lung decreased significantly by 3.3-fold from 52.05 ± 16.11 to 15.54 ± 5.36 arbitrary units (AU) (mean ± SEM) (*n *= 22, *P *< 0.01). The left lung sound amplitude decreased by 2.4-fold from 28.42 ± 11.28 to 11.69 ± 3.15 AU (mean ± SEM) (*n *= 22, *P *> 0.01). The flow rate (measured by the VRI D-lite flow meter) of both lungs increased significantly after secretion removal (*n *= 22, *P *< 0.01). See Figure 1.

## Conclusions

The finding that the VRI signal amplitude decreased after a suction procedure in ventilated patients suggests that secretions are usually noisy. This effect was more pronounced on the right side probably due to expected more efficient secretion removal. We suggest that effective removal of secretions may be inferred by a combination of a decrease in VRI signal coupled with an increase in air flow rate.
